# Dielectrophoresis Separation of Platelets Using a Novel Zigzag Microchannel

**DOI:** 10.3390/mi11100890

**Published:** 2020-09-25

**Authors:** Yanfang Guan, Yansheng Liu, Hui Lei, Shihua Liu, Fengqian Xu, Xiangxin Meng, Mingyang Bai, Xiaoliang Wang, Gexuan Yang

**Affiliations:** School of Electromechanical Engineering, Henan University of Technology, Zhengzhou 450001, China; liu1628822788@163.com (Y.L.); leihui@haut.edu.cn (H.L.); liugd2020@haut.edu.cn (S.L.); xufengqian1995@163.com (F.X.); mengxiangxin0717@126.com (X.M.); bmy0730@163.com (M.B.); xiaolaingwang@126.com (X.W.); hgdygx@163.com (G.Y.)

**Keywords:** zigzag-shape microchannel, Red blood cells (RBCs), platelets, dielectrophoresis

## Abstract

Platelet separation and purification are required in many applications including in the detection and treatment of hemorrhagic and thrombotic diseases, in addition to transfusions and in medical research. In this study, platelet separation was evaluated using a novel zigzag microchannel fluidic device while leveraging a dielectrophoresis (DEP) electric field using the COMSOL multiphysics software package and additional experimentation. The zigzag-shaped microchannel was superior to straight channel devices for cell separation because the sharp corners reduced the required horizontal distance needed for separation and also contributed to an asymmetric DEP electric field. A perfect linear relationship was observed between the separation distance and the corner angles. A quadratic relationship (*R^2^* = 0.99) was observed between the driving voltage and the width and the lengths of the channel, allowing for optimization of these properties. In addition, the voltage was inversely proportional to the channel width and proportional to the channel length. An optimal velocity ratio of 1:4 was identified for the velocities of the two device inlets. The proposed device was fabricated using laser engraving and lithography with optimized structures including a 0.5 mm channel width, a 120° corner angle, a 0.3 mm channel depth, and a 17 mm channel length. A separation efficiency of 99.4% was achieved using a voltage of 20 V and a velocity ratio of 1:4. The easy fabrication, lower required voltage, label-free detection, high efficiency, and environmental friendliness of this device make it suitable for point-of-care medicine and biological applications. Moreover, it can be used for the separation of other types of compounds including lipids.

## 1. Introduction

The rapid separation and purification of biological particles have attracted considerable research attention recently due to their value in research, especially in medical diagnosis and environmental detection applications [1,2,3]. Centrifugation [4,5] is the most frequently used technology for separating biological particles. However, centrifugation requires professional equipment, laboratory facilities, and operators, while also being expensive and not suitable for point-of-care testing (POCT) [6,7]. Consequently, methods to separate biological particles have been extensively explored, with microfluidic technology [8,9,10] emerging as a promising means for separating particles.

Microfluidics is a simple, low-cost, and easy-to-operate technology compared with traditional macroscale technology that requires large-scale operation equipment and a suitable laboratory facility. The principle of separation in microfluidic systems operates via well-designed microchannels and precisely controlled external physical separation methods that utilize acoustic fields [11,12], fluorescence activation [13,14], optical filing [15,16], magnetic activation [17,18], and electrical fields [19,20,21]. Shamloo et al. [11] simulated the separation of blood cells with acoustic fields, while evaluating the most efficient and low-cost separation devices by altering factors of acoustic field devices. In addition, Rosental et al. [14] demonstrated and developed a method to isolate different cell populations in corals and other cnidarians using fluorescent activated cell sorting (FACS) technology, which is an advanced cell sorting technique. Further, Tamura et al. [15] used optical cell separation technology to separate and classify heterogeneous cancerous cells based on morphology, confirming the feasibility of optical technology in cancer cell manipulation.

Dielectrophoresis (DEP) utilizes different charge characteristics among cells to achieve cell sorting and differential identification without relying on immunochemistry, making it useful for most cell sorting applications and rendering it different than other previously developed methods [21]. DEP leverages the differential dielectric responses in electric fields of particles with different structures and materials to achieve separation without destroying particle structures. The principle of DEP relies on different particles being separated by different forces in an inhomogeneous electric field [22]. Positive and negative electrodes are first applied to the sidewalls of separation devices. Different particles or cells then move toward different areas based on dielectrophoretic forces resultant from the asymmetric electric field (i.e., DEP). The unique features of the method make it simple, low-cost, and highly efficient [23]. Huang et al. [24] used DEP to separate monocytic cells from T cells, achieving separation efficiencies as high as 95%. In addition, Rahmani et al. [25] enriched biological particles by using a contactless DEP device, achieving enrichment rates of about 86%. Further, Liao et al. [26] separated plasma and blood cells from a whole blood sample (1 μL) using DEP, achieving a separation efficiency of 90%.

The measurement of platelet concentrations in blood is important for detecting many types of diseases. Low concentrations of platelets can lead to hemorrhage, and high concentrations can lead to thrombosis, stroke, and other diseases [27]. Thus, platelet separation using DEP devices have been proposed. For instance, Pommer et al. [28] used a two-stage dielectrophoresis-activated cell sorter (DACS) chip to separate platelets from blood. The electrodes were arranged on both sides of the device, and the researchers achieved 95% platelet purity. Piacentini et al. [29] also used rectangular electrodes arranged on a single side to achieve highly efficient platelet separation (98%) in microfluidic channels with lower driving voltages.

In this study, platelets and red blood cells (RBCs) were separated by DEP using a novel zigzag-shaped microchannel. Moreover, a unilateral zigzag-shaped arrangement was proposed in order to achieve a uniform distribution of potential in microfluidic chip channels, which represents a novel means to replace traditional DEP devices by arranging electrodes on double-layers with rectangular or semicircular configurations [28,29,30,31], leading to more efficient separation. Compared with electrode configurations in other channels, zigzag channels require electrode configurations around the corners of the channel, minimizing the negative effects of electrode slots on particle movement. Thus, particles can flow more smoothly and efficiently in the new devices, reducing particle loss rates. Moreover, zigzag channels represent deformed serpentine channels that more efficiently separate particles due to the influence of inertial forces [32,33,34]. An interlaced zigzag channel structure was implemented in a fabricated device and the COMSOL multiphysics simulation software package was used to arrange the electrodes on one side of the channel to form an uneven electric field distribution and achieve better particle separation. Lastly, the structure of the zigzag channel was further evaluated to compare particle separation characteristics for different structures and the effects of various factors on particle separation in order to achieve optimal cell separation.

## 2. Materials and Methods

### 2.1. Dielectrophoresis Theory

In this study, platelets and RBCs were regarded as spherical particles since the concentrations of white blood cells were very low and could be considered negligible. The time-averaged dielectrophoretic force (*F*_dep_) of spherical particles in an electric field is derived from the following equations [35]:

(1) Fdep=πr3ε1 Re[K(w)]∇|E|2

where *r* is the radius of the particle, $\varepsilon_{1}$ is the permittivity of the medium, and $\nabla {\left|E\right|}^{2}$ is the gradient of the electric field intensity squared. *K* is the Clausius-grope factor, namely CM factor. *K* is calculated from Equation (2) in the AC electric field:

(2) $$K=\frac{\tilde{\varepsilon_{2}}-\tilde{\varepsilon_{1}}}{\tilde{\varepsilon_{2}}+2\tilde{\varepsilon_{1}}}$$

where $\tilde{\varepsilon_{2}}$ and $\tilde{\varepsilon_{1}}$ are the complex permittivity of the particle and medium with the AC electric field, which may be calculated with the follow Equation (3):

(3) $$\tilde{\varepsilon_{i}}=\frac{\varepsilon_{i}-j\sigma_{1}}{w}$$

where σ is the conductivity, ω is the angular frequency, and *i* = 1, 2. When the particle is more prone to polarization than the medium (*Re* (*K*) > 0), the particle will generate a positive dielectric force (pDEP). If the medium is more susceptible to polarization than the particles (*Re* (*K*) < 0), a negative dielectric force (nDEP) will be produced.

### 2.2. Initial and Boundary Conditions

**Figure 1:**
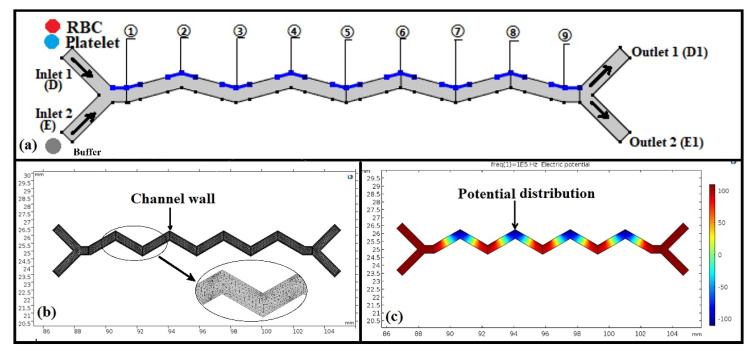
Initial and boundary condition settings for the zig-zag microchannel. (**a**) Inlet, outlet, and position of the potential in the microchannel. (**b**) The 2D finite element analysis (FEA) mesh. (**c**) Distribution of the non-uniform electric field by dielectrophoresis.

The zigzag-shaped microchannel used for platelet separation is shown in Figure 1a. A no-slip boundary condition was imposed between the fluids and channel walls. Fully developed flow conditions were applied at the D1 and E1 outlets using the COMSOL software package. In addition, alternating-current electric fields (1 × 10^−3^ Hz of driving frequency), creeping flow, and particle tracing for fluid flow models were used to promote separation. Released particle density was set as 1050 kg/m^3^. Inlet D was filled with RBCs and platelet microparticles. Inlet E was filled with phosphate buffer saline diluted in a sucrose solution to achieve a conductivity of 55 mS/m. Inlets D and E were set with different flow velocity ratios (1:4, 1:3, 1:2, and 1:1). The potential, as indicated in blue in Figure 1a, was loaded at the corner of the zigzag microchannel, where 1, 3, 5, 7, and 9 indicate the positive electrodes (+V) and 2, 4, 6, and 8 indicate the negative electrodes (−V). A rebound condition was used between the particles and the wall. To complete the simulation, the velocity field and DEP modules were added to the finite element analysis (FEA) module. The simulation mesh is shown in Figure 1b. The calculated electric field distribution in the zigzag microchannel was non-uniform, as shown in Figure 1c, making it suitable for the separation of particles with different sizes by DEP.

### 2.3. Fabrication of the Microfluidic Chip

**Figure 2:**
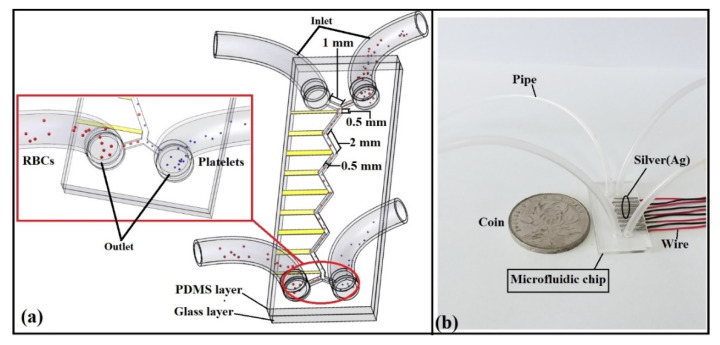
Diagram of the dielectrophoresis-based microfluidic chip for red blood cell (RBC) and platelet separation. (**a**) Structural parameters of the microfluidic chip and (**b**) image of the microfluidic chip after sealing.

The fabricated microchip included two layers (15 mm × 30 mm × 1 mm), as shown in Figure 2. The upper layer was equipped with two inlet/outlet pipes with 2 mm outside diameters and was fabricated using polydimethylsiloxane (PDMS) using reverse molding technology. The lower layer was designed with a zigzag-shaped microchannel (corner angle = 120 gz, channel width = 0.5 mm, channel depth = 0.3 mm, and channel length = 17 mm) and inlet/outlet holes using the CAD software package. The microchannel and holes were subsequently carved to a depth of 0.3 mm using a laser engraving machine. The silver electrode pattern (Ag, 300 nm) was deposited on the lower glass wafer using traditional lithography. Wires were arranged at the sidewall positions. Finally, the two layers were irreversibly bonded after placing the upper layer of PDMS under a mercury lamp for 30 min, as shown in Figure 2b.

### 2.4. Materials and Experimental Setup

The experiments utilized a laser engraving machine (MUV-E-A, Yueming Laser, Guangdong, China), an electron beam evaporator (E-beam VT1-10CE, ULVAC, Shanghai, China), an ABM semi-auto mask aligner and UV exposure system (ABM/6/350/NUV/DCCD/BSV/SA, ABM, Chicago, IL, USA), a syringe pump (LSP01, Baoding, China), a signal generator (DG1022, Rigol, Zhengzhou, China), a Charge-coupled Device (CCD)and microscope (Obvious Ltd., Co., Guangzhou, China), PDMS (Sylgard 184, Dow Corning, Midland, MI, USA), and a flow cytometer (FACS CALIBAR, BD Biosciences, Shanghai, China).

Blood was collected from healthy volunteers and a sodium citrate solution was added to prevent blood clotting. The blood was subsequently placed in a centrifuge tube and centrifuged for 10 min at 1000 rpm. The upper supernatant in the tube (containing mostly platelets) and the lower portion (primarily containing RBCs) were mixed and diluted at a 1:10 ratio with normal saline to obtain the experimental samples. Flow cytometry was used for cell selection, with 10,000 RBCs and 10,000 platelets used for the experimental samples. Phosphate buffer saline (PBS) was used as the working solution to adjust the conductivity of the samples to 55 mS/m in order to maintain the osmolarity. All experiments were approved by the ethical committee at the Henan University of Technology, and were performed in compliance with the ethical policy for use of human subjects according to the national guidelines of China. Informed consent was obtained for all experimentation with human subjects.

**Figure 3:**
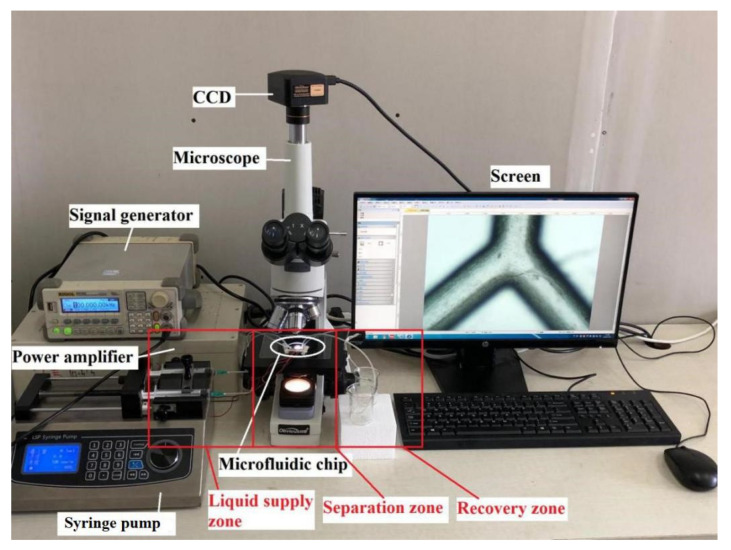
Experimental platform for platelet separation.

The experimental setup was divided into three zones for liquid supply, separation, and recovery (Figure 3). The sample and buffer solution (PBS) were supplied by an injection pump at speeds of 200 and 800 μm/s, respectively, from inlets D and E, respectively. The fluid field was observed microscopically and with the CCD camera. Samples in the recovery zone were evaluated with flow cytometry to determine separation efficiency.

## 3. Results and Discussion

### 3.1. Structural Parameters and Analysis of Influential Parameters

DEP occurs when dielectric particles are subjected to an uneven electric field and is widely used in biomedical equipment including biosensors, diagnostic machines, particle manipulation and filtration (sorting) devices, and particle assembly. Here, we evaluated the use of a zigzag flow channel for DEP applications. Several putative influential factors are involved in DEP separation and were evaluated individually.

#### 3.1.1. Influence of Corner Angles

**Figure 4:**
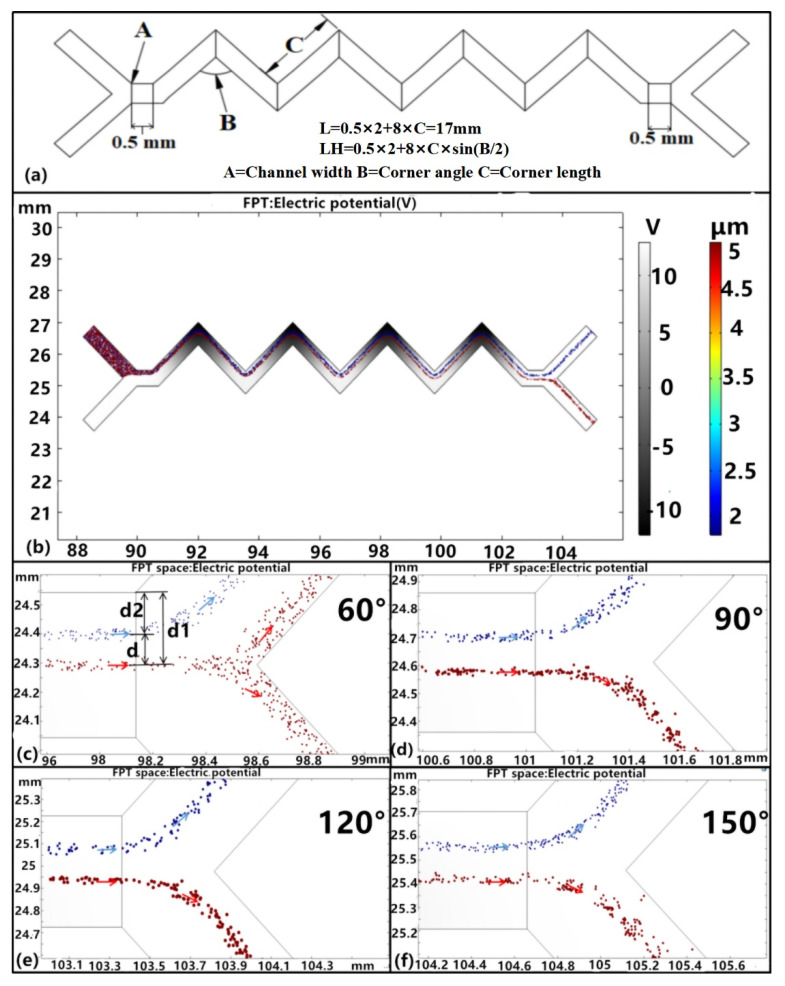
Structural parameters and platelet separation analysis. (**a**) The structural parameters of the zigzag-shaped microchannel. (**b**) Cell separation resulting from the zigzag channel. (**c**–**f**) Analysis of the separation distance at corner angles of 60°, 90°, 120°, and 150°, respectively, blue color arrows mean the flow trajectory of the platelet and red color arrows mean the RBC flow trajectory.

To evaluate the influence of corner angles on platelet sorting, other structural parameters of the zigzag microchannel were fixed (Figure 4a). The channel width (A) was set to 0.5 mm, the corner length (C) to 2 mm, and corner angles of 60°, 90°, 120°, and 150° were evaluated (B). The velocities at inlets D and E were set at 200 and 800 μm/s, respectively, and the driving voltage and frequency as 20 V and 1 × 10^−3^ Hz.

The particles were separated in the zigzag channel (Figure 4b), although this was not possible when the corner angle was 60° (Figure 4c). Further, some of the RBCs were lost with the platelets because of small angles, indicating that particles are more likely to mix rather than separate in passages with too small of angles [36]. In contrast, the separation of platelets and blood cells was achieved when the corner angle was increased to 90° (Figure 4c). Moreover, larger corner angles produced better separations based on the separation distance, wherein the distance between the platelets and RBCs was equal to d, the distance between the RBCs and the voltage-applied wall side was equal to d_1_, and *d* = *d_1_* − *d*_2_ in Figure 4b. Larger angles changed the distribution of the electric field, and the different DEP forces induced by the non-uniform electric field then acted on the platelets and RBCs, leading to total separation (Figure 4c–e). Indeed, a perfect linear relationship (*R^2^* = 1) was observed between the corner angle and the separation distance d, as shown in Figure 4f.

**Figure 5:**
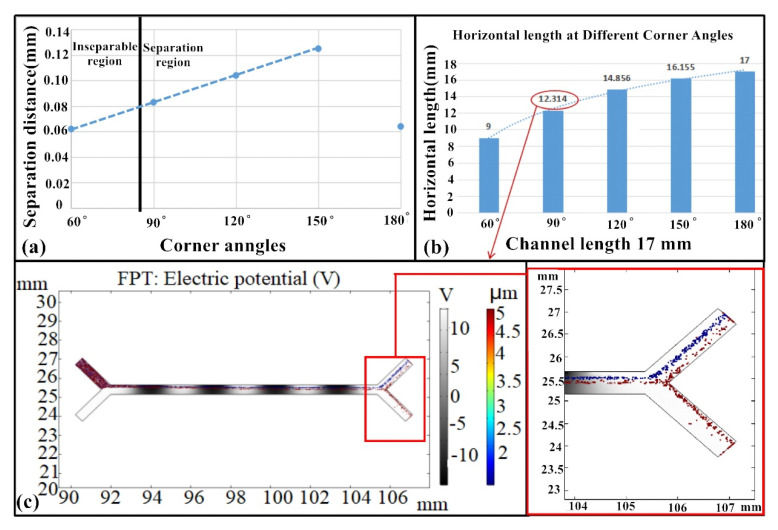
Comparison between straight and zigzag-shaped microchannel. (**a**) Linear relationship between separation distance and corner angles. (**b**) Comparison of the horizontal and spread lengths for different corner angles. (**c**) Diagram showing the inseparability of platelets from RBCs in the straight microchannel for an *L*_H_ = 12.314 mm (compared against B = 90°). All experiments were replicated with *n* = 5 and values are shown as mean ± SD.

Larger corner angles did not, however, guarantee better separation (Figure 5a,b) and minimizing space is one of the main advantages of the zigzag microchannel. For example, total platelet separation was not achieved using a straight microchannel with an *L* = 12.314 mm (Figure 5c). However, separation was achieved when the corner angle (B) was 90° with an *L*_H_ = 12.314 mm (Figure 5a). Note that the arrows in Figure 5b point to Figure 5c, indicating that the same horizontal distance with a 90° corner angle was equivalent to the straight channel in Figure 5b in order to compare the two scenarios. Importantly, the zigzag microchannel conserved space compared with the straight microchannel, while achieving complete separation, with the latter requiring a longer distance to separate the platelets (Figure 5a). Moreover, an electric field can be easily applied to the zigzag-shaped microchannel, and cells do not become trapped in the channel due to construction considerations. Rather, the cells move up and down, accelerating cell separation, shortening separation time, and enhancing separation efficiency (discussed below).

#### 3.1.2. Influence of Channel Width and Length

**Figure 6:**
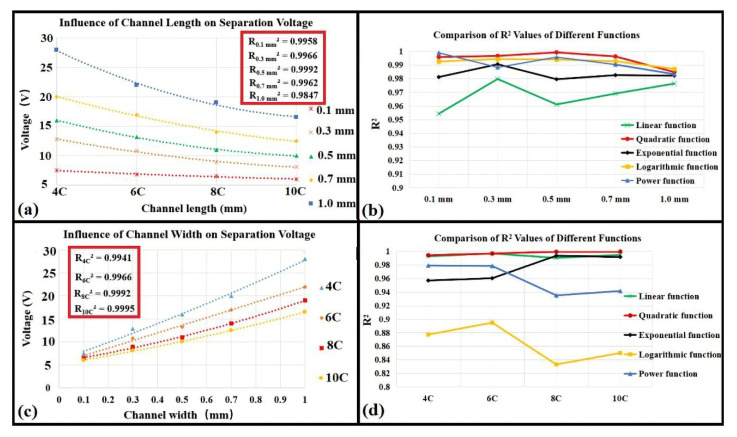
Influence of channel length and width on separation voltage. (**a**) The relationship between channel length and driving voltage. (**b**) Correlation coefficients (*R*^2^ values) of fitted functions for different channel lengths. (**c**) Proportional relationships between channel widths and driving voltages. (**d**) Correlation coefficients (*R*^2^ values) for different fitted functions for different channel widths. All experiments were repeated with *n* = 5, and values are shown as mean ± SD.

The corner angle affected the distribution of the electric field, while channel width and length affected the separation distance of platelets and RBCs. To compare the effects of the width and length relative to the voltage, the corner angle and inlet velocity ratios were fixed at 120° and 1:4, respectively. Linear, quadratic, exponential, logarithmic, and power function fitting of the driving voltage were calculated separately as a function of the channel width and length (Figure 6). The driving voltage was considered as the minimum separation voltage, wherein the separation of the platelets and RBCs was obtained once the voltage was larger than this value based on simulations. Comparison of correlation coefficient (*R*^2^) values for various models indicated that the quadratic functions best expressed the relationships between width, length, and voltage, as exemplified by *R*^2^ values that were nearly all > 0.99 (Figure 6b,d). Furthermore, the length of the channel was inversely proportional to the voltage (Figure 6a,c) and the width was proportional to the voltage. Specifically, a larger width and smaller length corresponded to a greater driving voltage. Thus, when fabricating microfluidic chips, the channels should be as narrow as possible while satisfying process requirements, which ultimately saves space and reduces energy losses while also achieving better separation.

#### 3.1.3. Influence of the Velocity Ratio

**Figure 7:**
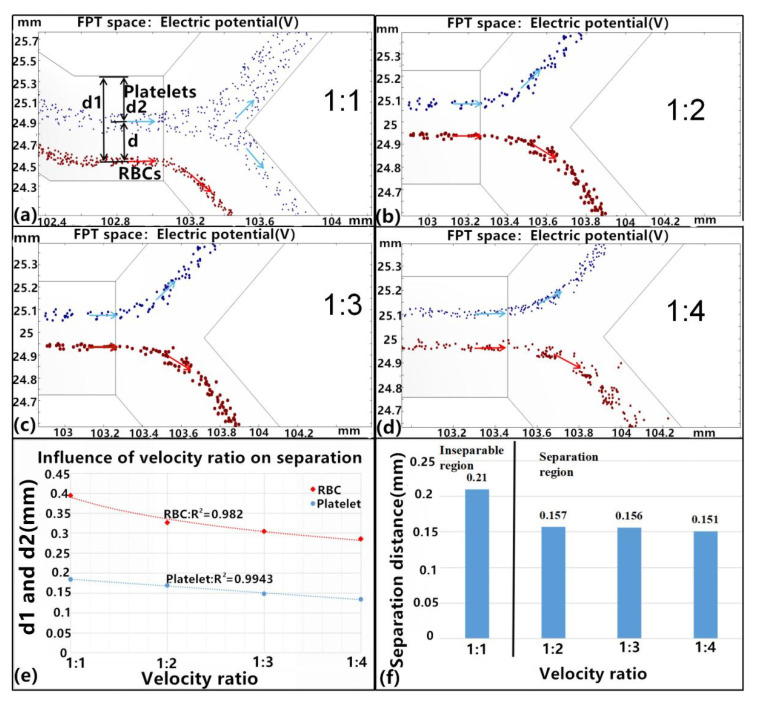
Separation conditions in response to the velocity ratio. Separation results are shown when using velocity ratios of (**a**) 1:1, (**b**) 1:2, (**c**) 1:3, and (**d**) 1:4, blue color arrows mean the flow trajectory of the platelet and red color arrows mean the RBC flow trajectory. (**e**) The relationship between separation distances d_1_ and d_2_ and different velocity ratios. (**f**) Comparison of platelet and RBC separation distances. All experiments were conducted with *n* = 5 and values are shown as mean ± SD.

To examine the influence of inlet velocity on particle flow within the channel, velocity ratios of 1:1, 1:2, 1:3, and 1:4 were used between inlets D and E, and the simulated separation effects were analyzed (Figure 7a–d). In the simulations, the structural parameters were fixed to ensure that the particles were separated with a corner angle = 120°, length = 8C, width = 0.5 mm, and driving voltage = 20 V. The velocity at inlet D was set as 200 μm/s and the velocity at inlet E was varied to achieve the desired ratio. When the velocity ratio was 1:1, platelets were not fully separated (Figure 7a). When the ratio was increased to 1:2, the platelets and RBCs separately flowed out from the two outlets. Moreover, the separation distance d_2_ decreased as the velocity ratio increased (Figure 7e), as modeled by a quadratic function that captured distance variation. Thus, the velocity ratio significantly affected the distance d1, but had a small influence on the distance d (Figure 7f). Consequently, to achieve complete separation of platelets from blood samples, the buffer velocity should be higher than that of the blood sample when using the same driving voltage in order to ensure that the sample is closer to the side of the electrode. If the above is not achieved, particle separation will be affected.

#### 3.1.4. Influence of Driving Voltage

**Figure 8:**
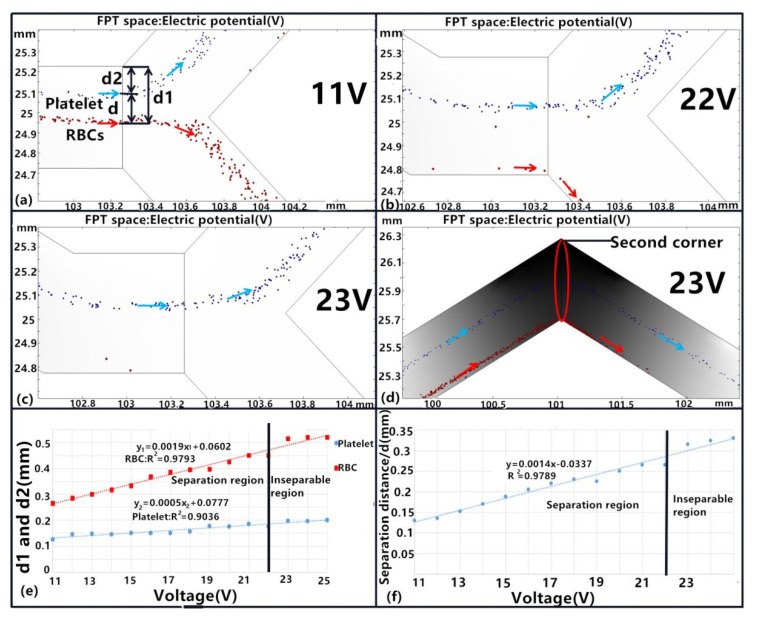
Effects of the driving voltage on platelet separation. (**a**–**d**) Separation under different driving voltages of 11, 22, and 23 V, Blue color arrows mean the flow trajectory of the platelet and red color arrows mean the RBC flow trajectory. (**e**) Comparison of platelet and RBC separation distances with different driving voltages. (**f**) The relationship between separation distance and driving voltages. All experiments were conducted with an *n* = 5, and values are shown as mean ± SD.

As shown previously, the corner angle, channel width, and length of separation devices can directly affect the distribution of electric fields, thereby affecting the separation of particles with different DEP forces. Consequently, the driving voltage is critical for platelet or other types of separation, as shown by Equation (1). The platelets were separated normally when the driving voltage ranged between 11 and 22 V (Figure 8a,b) and the structural parameter values of the zigzag microchannel were the same as described in Section 3.1.3. However, when the voltage was higher than 22 V or lower than 11 V, platelets were not separated from the blood samples and the platelets flowed out from the upper outlet (Figure 8c). Further, there was no RBC flow out of both outlets when using a 23 V driving voltage, wherein the RBC remained on the lower wall when moving along the zigzag channel to the second corner angle. These activities can be explained by the relationship between DEP forces and the electric field, as given by Equation (1). Thus, the distances d_1_ and d_2_ increased with the driving voltage (Figure 8e). The higher the voltage, the greater the separation effect on separation distance, wherein a voltage of 22 V was the critical threshold value for platelet separation from blood samples. Overall, these results indicate that voltage had the most significant effect on particle separation in the dielectric field. Consequently, the critical separation point of particles can be achieved by adjusting the voltage range for different microchannel structures.

#### 3.1.5. Influence of Driving Frequency

**Figure 9:**
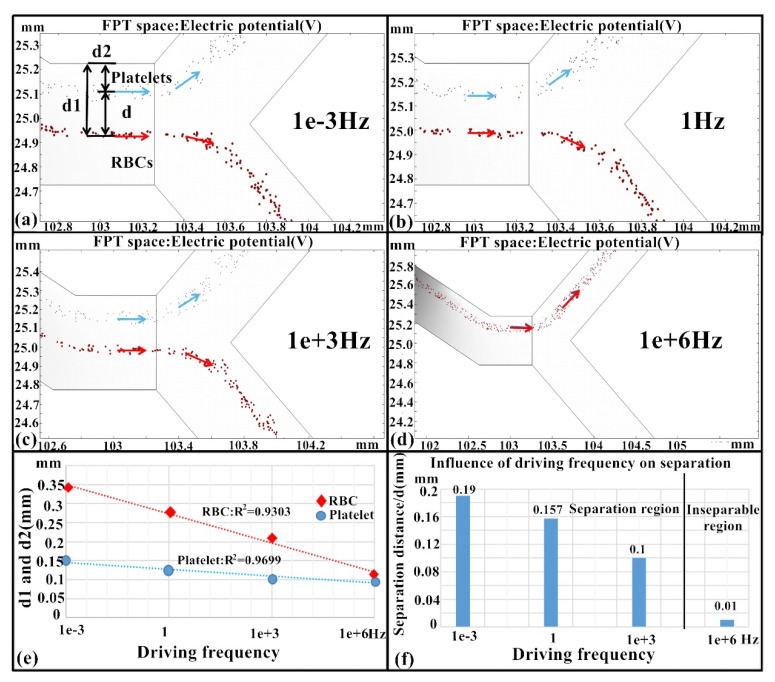
Effects of the driving frequency on platelet separation. (**a**–**d**) Separation under different driving frequencies of 1 × 10^−3^ Hz, 1 Hz, 1 × 10^3^ Hz, and 1 × 10^6^ Hz, blue color arrows mean the flow trajectory of the platelet and red color arrows mean the RBC flow trajectory. (**e**) Comparison of platelet and RBC separation distances with different driving frequencies. (**f**) The relationship between separation distance and driving frequencies. All experiments were conducted with an *n* = 5, and values are shown as mean ± SD.

Figure 9 shows the separation performance with respect to the driving frequency at a constant driving voltage of 12 V and a 1:4 velocity ratio between RBCs and platelets. The results indicated that the separation performance was inversely proportional to the driving frequency (Figure 9f). That is, lower driving frequency like 1 × 10^−3^ Hz led to a better separation effect as indicated by a larger separation distance and the effect diminished as frequency increased until a failure was obtained at 1000 kHz.

#### 3.1.6. Influence of Cell Size

**Figure 10:**
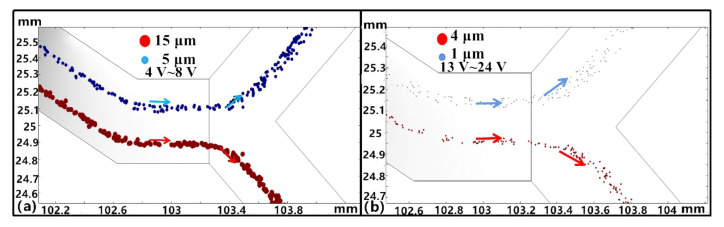
Separation conditions when using different cell sizes. (**a**) Separation of RBCs and platelets with maximum sizes of 15 and 5 μm, respectively. (**b**) Separation of RBCs and platelets with minimum sizes of 4 and 1 μm, respectively. All experiments were conducted with an *n* = 5, and values are shown as mean ± SD.

When the RBCs and platelets flowed in the microchannel, cell deformation could occur due to the electric field and capillary forces [37]. To account for this, possible cell sizes with minimum and maximum values of 4 and 15 μm for RBC diameters, respectively, and 1 and 5 μm for platelet diameters, respectively, were used to model separation while keeping the other parameters the same as described in Section 3.1.4. Platelet separation with various cell sizes could be achieved by adjusting the driving voltage (Figure 10). Thus, if the cell diameters are large, a lower voltage should be used. In contrast, a higher voltage should be used for the separation of small-sized cells. For example, the cells become larger and smaller in the case of the two cell sizes, with their respective voltage ranges being 4–8 V and 13–24 V, respectively (Figure 10a,b). The large cells were more easily separated with low voltage and low voltage regulation. When the deformation of particles was small, the voltage needed to be adjusted within the normal range (Figure 10a). Larger particle deformations required smaller voltage values (Figure 10b). Thus, these results also show that relatively large particles were more suitable for DEP separation when the channel structure was consistent and energy loss was reduced.

### 3.2. Experimental Analysis of Platelet Separation Efficiency

**Figure 11:**
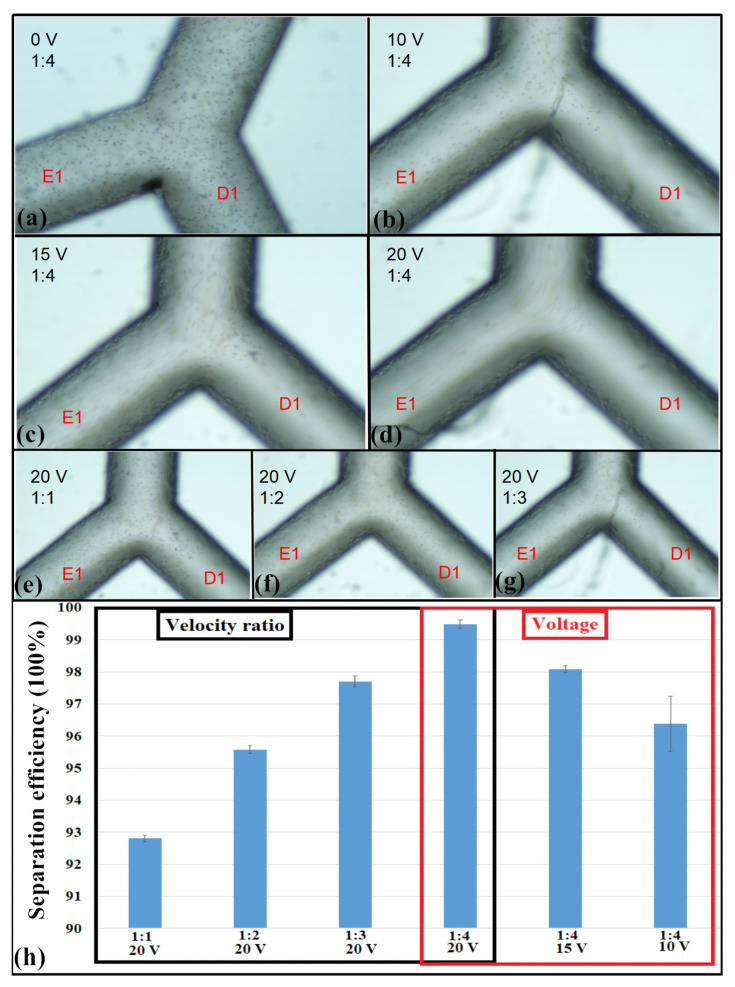
Experimental platelet separation using different driving voltages and velocity ratios based on dielectrophoresis (DEP) forces in zigzag-shaped microchannels. (**a**–**d**) Platelet separation under voltages of 0 V, 10 V, 15 V, and 20 V, respectively. (**e**–**g**) Separation effects at velocity ratios of 1:1, 1:2, and 1:3. (**h**) Comparison of separation efficiency using different voltages and velocity ratios. All experiments were conducted with an *n* = 5, and values are shown as mean ± SD.

To further verify the use of zigzag microfluidic chips on cell separation, a practical experiment was conducted. Samples containing platelets, RBCs, and PBS solutions were injected into a microchannel from inlets D and E at a velocity ratio of 1:4 (200 and 800 μm/s, respectively) using a syringe pump. To evaluate the effect of different driving voltages on separation, several voltages (0, 10, 15, and 20 V) and velocity ratios (1:1, 1:2, 1:3, and 1:4) were used (Figure 11a–g). No separation of platelets and RBCs occurred at a voltage of 0 V, because a DEP force was not present in the microchannel (Figure 11a). As voltage increased to 10 and 15 V, platelet and RBC separation occurred, and many RBCs flowed out far from the electrode and the exit E_1_ (Figure 10b,c). Increased voltage to 20 V resulted in perfect platelet separation (Figure 11d), with almost no RBCs observed in D_1_. The same result occurred with velocity ratios of 1:1, 1:2, 1:3, and 1:4. Further, when the inlet velocity ratio was increased from 1:1 to 1:4, better platelet separation occurred (Figure 11e–g). The platelet separation efficiency was calculated from counting the number of cells in the samples and from the outlet, as follows:

Separation efficiency = (Cell numbers in outlet D1/Cell numbers in sample) × 100%(4)

A maximum separation efficiency of 99.4% was achieved using a driving voltage of 20 V and a velocity ratio of 1:4 (Figure 11h). Thus, complete separation of platelets and RBCs was achieved. A small number of false detections may have occurred due to cell deformation, cell death, or parts of cells sticking to the walls during separation. These results agreed with the simulation analysis described in Section 3.1.3 and Section 3.1.4. Higher driving voltages and velocity ratios resulted in better separation efficiency. Thus, the zigzag-shaped microchannel achieved better separation effects if the driving conditions were adjusted to optimal values. No electrode slot was needed to achieve the DEP force because the special zigzag-shaped structure was complete and smooth, preventing a possible loss of particles trapped in the slot. Smaller channel widths resulted in smaller required voltages and better separation effects.

In addition to being used to achieve high-efficiency platelet separation, the new zigzag-shaped DEP device can also be used to separate other types of cells and non-cellular particles. Moreover, the device could be used for particle purification via altering the structural parameters to proper values. Furthermore, the lower required driving voltage, lack of pollution output, and simplicity of operation will render this new device popular for point-of-care medicine and biology.

## 4. Conclusions

In this study, we proposed a new zigzag-shaped microchannel configuration to achieve platelet separation through asymmetrical electric fields created by DEP forces that influenced cell motion. Structural parameters that influenced separation including the corner angle, channel width, channel length, driving voltage, velocity ratio, and cell size were analyzed using the COMSOL software package, leading to the identification of optimal structural parameters. In contrast to the electric fields distributed by two electrodes in traditional DEP, the proposed device used a zigzag scattered structure to arrange the electrodes, allowing optimal separation of RBCs and platelets. In the configuration, RBCs and platelets were allowed to flow smoothly throughout the whole channel, unlike with traditional electrode slots that can trap particles. When the corner angles were between 90 and 150°, the separation distance of the particles and the sizes of the corners were perfectly linearly related (R^2^ = 1), while particles could not be separated at angles of 60° or less. Moreover, the length and width of the channel significantly affected the DEP electric field distribution, with a quadratic relationship best describing their relationships. The voltages and velocity ratios of the inlets affected the movement of particles in the channel, wherein velocity ratios were required to be in a certain range, otherwise particles could not normally flow from the inlet and outlet. A suitable velocity ratio range was observed as 1:2–1:4, and the velocity of the buffer should be greater than that of the sample. The voltage also affected the separation of particles and was proportional to the separation distance. The voltage range at which separation occurs can be determined based on the separation structure. Finally, perfect separation of platelets and RBCs (separation efficiency of almost 99.4%) was achieved on a microfluidic chip using a voltage of 20 V. Thus, the DEP device using a zigzag microchannel exhibited high efficiency and practical operability. Furthermore, this device can be used to separate other types of cells or particles and demonstrates a new conceptual model for particle separation applications in medical biology due to its convenience, high efficiency, and lack of environmental pollution.
